# Biophysical Modulation of Mesenchymal Stem Cell Differentiation in the Context of Skeletal Repair

**DOI:** 10.3390/ijms23073919

**Published:** 2022-04-01

**Authors:** Clark T. Hung, Jennifer Racine-Avila, Matthew J. Pellicore, Roy Aaron

**Affiliations:** 1 Department of Biomedical Engineering, Columbia University, New York, NY 10032, USA; cth6@columbia.edu (C.T.H.); mjp2264@columbia.edu (M.J.P.); 2 Department of Orthopedic Surgery, Columbia University, New York, NY 10032, USA; 3 Department of Orthopedics, Alpert Medical School of Brown University, Providence, RI 02905, USA; jracine@lifespan.org

**Keywords:** stem cells, differentiation, osteogenesis, chondrogenesis

## Abstract

A prominent feature of the skeleton is its ability to remodel in response to biophysical stimuli and to repair under varied biophysical conditions. This allows the skeleton considerable adaptation to meet its physiological roles of stability and movement. Skeletal cells and their mesenchymal precursors exist in a native environment rich with biophysical signals, and they sense and respond to those signals to meet organismal demands of the skeleton. While mechanical strain is the most recognized of the skeletal biophysical stimuli, signaling phenomena also include fluid flow, hydrostatic pressure, shear stress, and ion-movement-related electrokinetic phenomena including, prominently, streaming potentials. Because of the complex interactions of these electromechanical signals, it is difficult to isolate the significance of each. The application of external electrical and electromagnetic fields allows an exploration of the effects of these stimuli on cell differentiation and extra-cellular matrix formation in the absence of mechanical strain. This review takes a distinctly translational approach to mechanistic and preclinical studies of differentiation and skeletal lineage commitment of mesenchymal cells under biophysical stimulation. In vitro studies facilitate the examination of isolated cellular responses while in vivo studies permit the observation of cell differentiation and extracellular matrix synthesis.

## 1. Introduction

**Figure 1:**
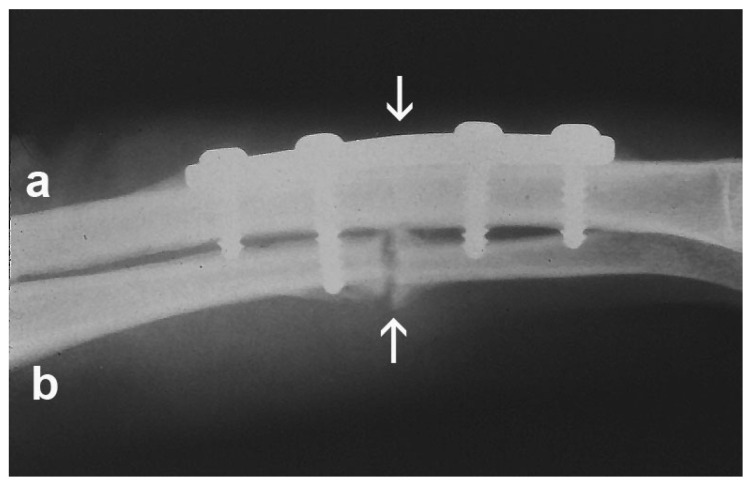
Fracture healing in two different mechanical strain environments. Fracture sites are indicated by arrows. The radius (**a**) fixed with a rigid plate, and experiencing minimal mechanical strain, is healing by direct osteonal repair. The ulna (**b**), which is in a more flexible mechanical environment, and is experiencing motion and strain, is healing by endochondral bone formation. For both the radius and the ulna, the ECM and gene expression of the repair are being regulated by the biophysical environments. Reproduced with permission from Aaron RK, Bolander ME (eds). Physical Regulation of Skeletal Repair. Rosemont, IL: American Academy of Orthopaedic Surgeons; 2005.

A notable feature of the skeleton is its plasticity in response to changing biophysical environments. Remodeling of the skeleton under high mechanical load offers the ability to develop strength through hypertrophy to withstand strain. Repair of the skeleton is greatly influenced by its strain environment. Figure 1 shows two fractures, the radius, and the ulna in the human forearm, healing under different biophysical conditions, and, in response to their strain environments, healing with very different physiologies, extracellular matrix (ECM) synthesis, and gene expression. This demonstrates in a physiologically relevant human model of skeletal repair, that the biophysical environment (mechanical in this case) can influence gene expression [1].

This review presents the influences of some biophysical forces on skeletal tissues and cells, notably skeletal-derived mesenchymal stem cells (MSCs), and emphasizes physically enhanced morphogenesis in the context of repair. The review takes a distinctly translational approach. In vitro and in vivo experiments provide complementary opportunities for examining the responses of mesenchymal cells. In vitro models permit the detailed examination of cellular responses including signal transduction, while in vivo models allow observation of cell differentiation in a developmental process such as endochondral bone formation. In describing together these cell and tissue responses to biophysical stimuli, the potential of how these responses translate to the clinic is pointed out. The review examines external electromechanical stimulation of MSCs from a particular point of view. Because of the intrinsic coupling of mechanical strain, fluid flow, and electrical phenomena, it is extremely difficult to isolate the effects of specific biophysical phenomena on cultured cells or tissues. The application of external electrical or electromagnetic fields, however, can explore the effects of electrical stimuli on proliferation and differentiation in the absence of mechanical loading. The focus is on modulation of MSC-mediated morphogenesis by external biophysical stimuli and the contributions of both in vivo models describing developmental processes and in vitro models describing cellular responses, and how they can be interpreted together to inform translational efforts and clinical applications, notably in the context of repair.

### 1.1. Influence of the Biophysical Environment and Cell Reception

**Figure 2:**
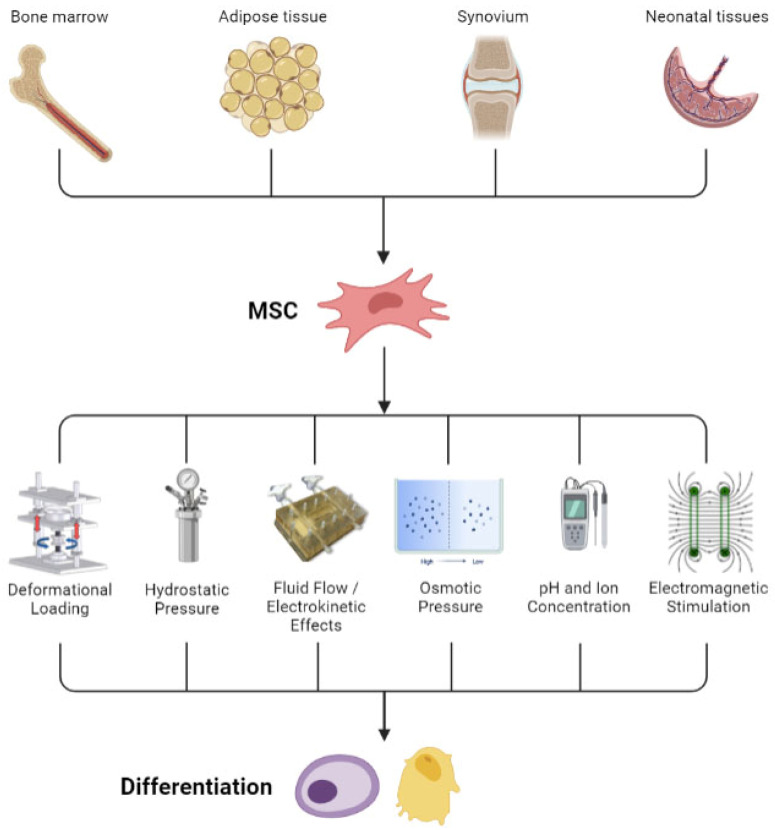
Representation of the harvest of MSCs from multiple tissue sources and biophysical stimulation of the cells to induce differentiation.

Skeletal tissues and MSCs exist in a biophysically rich microenvironment that provide cells with many mechanical, electrical, and biochemical cues. In turn, cells have evolved an array of organelles and transduction pathways for sensing and responding to physical signals to meet their physiological requirements. These mechanisms include direct mechanosensing, recognition of fluid flow patterns, and electrokinetic events by cell deformation, membrane shape changes, adhesion molecules, cytoskeletal signal transmission, and mechanosensitive transmembrane ion channels. Intracellular signaling pathways transduce the sensed signals, eliciting responses usually composed of the synthesis of ECM molecules to reinforce or repair local structures to meet the demands of the environment. Cells express genes that encode for structural and signaling molecules that remodel the ECM and provide paracrine signaling, respectively, ultimately leading to changes in cell proliferation, differentiation, and morphogenesis [2,3]. Expression of a chondrogenic or osteogenic phenotype by MSCs is characterized by the synthesis of characteristic ECM molecules including collagen, proteoglycan, fibronectin, laminin, and the receptor for hyaluronan and osteopontin (CD44) [4]. In this review, the effects of biophysical stimuli—notably, mechanical, electrical, and electromagnetic energy—on mesenchymal cells are emphasized while a redundancy of established descriptions of MSCs is avoided [5] (Figure 2).

### 1.2. Mechanical and Electrical Influences on Lineage Expression of MSCs

The adoption of specific lineages by MSCs is dependent, in part, upon the microenvironment and the cytokines to which MSCs are exposed. The mechanical properties of native tissue stem cell niches, in vitro substrates, and external mechanical stresses in vivo have major effects on MSC differentiation. MSCs adopt varying morphologies in response to microenvironments of varying mechanical properties and to external mechanical stresses. The activation of mechanosensitive membrane receptors and intracellular messengers, such as the mitogen-activated protein (MAP) kinases by mechanical environments, are important transduction pathways of mechanical signals that influence differentiation and phenotypic expression of MSCs [6]. The stiffness of tissue niches influences differentiation and lineage commitment of MSCs [7]. In vitro substrates have been used to mimic tissue stiffness, stimulate cell adhesion, and lineage commitment. External mechanical loading, particularly cyclic loading, and fluid shear flow, also influence lineage commitment of MSCs [8]. Changes in tissue stiffness and extrinsic mechanical loading generate fluid flow. Because tissue fluid, and many culture bioreactors, contain ion-rich fluid, in addition to hydrostatic pressure and shear stress, fluid flow establishes ion fluxes, streaming potentials and other electrokinetic phenomena to which MSCs are sensitive.

## 2. Response to Mesenchymal Cells to Biophysical Regulation and Mechanical Strain Associated Events In Vitro

In addition to biochemical regulation, biophysical signaling in response to hydrostatic pressure, fluid flow and accompanying shear stress, substrate strain and stiffness, substrate topography, and electromagnetic fields have induced chondrogenic and osteoblastic differentiation of MSCs in vitro [9]. Matrix elasticity has been shown to direct stem cell lineage specification. Culturing of MSCs on 2D substrates having stiffness of brain, skeletal muscle, and osteoid bone can confer neurogenic, myogenic, and osteogenic differentiation, respectively [7]. The ability of MSCs to sense matrix elasticity is related to their contractile behavior [7], where early changes in cytoskeletal contractility predict later stem cell fate decisions in single cells [10]. The topography of biomaterials modulated through roughness, patterns, and porosity are an efficient approach to control the fate of MSCs and the application of topography in tissue engineering [11]. The stress relaxation behavior of hydrogel scaffolds can modulate stem cell differentiation in 2D [12] and 3D [13].

Physiologic loading of the musculoskeletal system gives rise to strain-associated biophysical stimuli such as deformational loading, hydrostatic pressure, fluid flow, electrokinetic phenomena, and osmotic loading, that modulate cell activities responsible for tissue remodeling, repair, and maintenance [14]. Biomimetic stimuli, therefore, represent a complementary input to traditional chemical mediators to modulate MSC differentiation and function. These stimuli appear to induce lineage-specific responses; for example, applied hydrostatic pressure pushes chondrogenic differentiation [15,16], whereas tensile loading pushes tenogenic and osteogenic differentiation [17].

Cell Sensing-Biophysical Transduction: Primary cilia are sensory organelles that extend into the extracellular milieu and have been shown to play a critical role in chemical and mechanical induction of stem cell differentiation [18,19,20,21]. Changes in cell properties will influence their interaction with the environment. For instance, adipose-derived stem cell mechanical properties, as measured by atomic force microscopy, change with adipogenic and osteogenic differentiation [22]. For osteogenesis, cells typically become stiffer, whereas for adipogenesis, cells become more compliant [22]. In separate studies using micromolded elastomeric micropost arrays, traction force per cell has been shown to increase with MSCs subjected to osteogenic media, whereas it decreases with adipogenic media. These early changes in cytoskeletal contractility predicted later stem cell fate decisions in single cells [10]. Chondrogenesis induces intracellular changes that result in higher instantaneous and relaxed moduli as well as higher apparent viscosities [23]. The nucleus has also been reported to act as a mechanostat to modulate cellular mechanosensation during differentiation [24].

**Figure 3:**
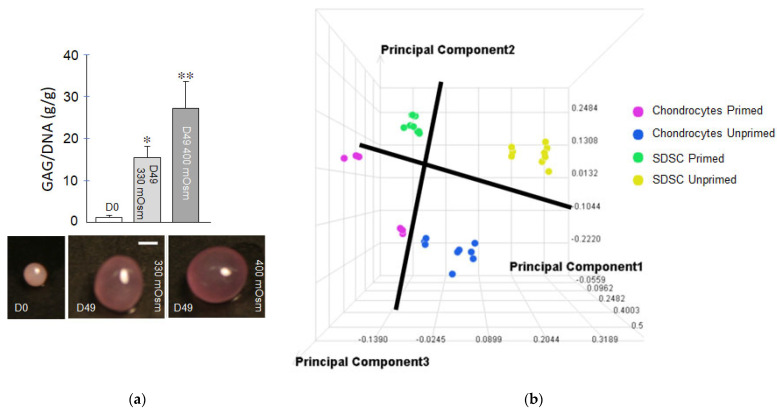
(**a**) Glycosaminoglycan (GAG) content of SDSC micropellet cultures was promoted by physiologic hypertonic media (400 mOsm) relative to isotonic media (330 mOsm), similar to that reported for SDSCs in 3D hydrogel culture [28]. * *p* < 0.05 vs D0; ** *p* < 0.05 vs other groups (n = 3–4/group). Scale bar: 1mm. (**b**) Principal component analysis of Z-score transformed intensity data processed by the Elucidator program for the 33 LC/MS chromatograms performed in a study [25] where canine SDSCs were primed in culture media containing (1 ng/mL TGF-β1, 5 ng/mL bFGF, and 10 ng/mL PDGF-BB). Each data point represents a chromatogram. Data from unprimed cells are found on the right side of the first principal component axis. Data from primed cells are grouped on the left side of that axis, suggesting an overall global effect of the growth factor priming. The distance between unprimed and primed chromatograms was greater for SDSCs than for chondrocytes, suggesting that priming yielded a more pronounced effect for SDSCs. Unpublished data from Dr. Hung’s laboratory.

Cell priming: Chemical and physical priming of cells during 2D expansion has subsequent impacts during MSC differentiation and ECM synthesis in 3D. During differentiation, specific media components [25] in conjunction with physical (e.g., osmotic) [26,27] loading of synovium-derived stem cells (SDSCs)—MSC-like cells residing in the synovial membrane—promote development of functional engineered cartilage [28,29], as shown in Figure 3a. Our studies show that chemical priming of SDSCs pushes their proteomic profile to that of articular chondrocytes, as shown in Figure 3b, fostering their subsequent cartilaginous tissue formation in culture [28,29]. Human MSC aggregates showed that both chondrogenic chemical priming and applied hydrostatic pressure in tandem during the priming period (21 days), followed by 21 days culture in osteogenic medium, accelerated the osteogenic potential of human MSCs [30].

Electric Fields: MSCs exhibit anodal migration in an applied direct current electric field (0.25–3 V/cm). This galvanotaxis is decreasingly observed from passage P3 to P10 without a negative impact on their osteogenic capacity (2 V/cm). The latter suggests that applied DC fields can be used to direct MSC migration for bone repair [31]. The galvanotaxis response may also serve as a tool for monitoring MSC differentiation. Early passage (P1–P2) SDSCs maintained in chondrogenic priming media exhibit anodal migration when subjected to applied DC electric field strength of 6 V/cm. By passage 3 and 4, these cells exhibited cell migration toward the cathode, as previously observed for terminally differentiated chondrocytes. This galvanotaxis response correlates with CD73 expression, a MSC cell surface marker, which decreases P3-P4. Only late passage cells (P4) were capable of developing cartilage-like tissue in 3D micropellet culture [32]. Pulsed electromagnetic fields (PEMFs) have been reported to stimulate osteogenic differentiation in human progenitor cells [33], as well as chondrogenesis and paracrine function of MSCs [34,35] as described further in the in vivo section.

Deformational Loading: Applied deformational loading of agarose-encapsulated MSC hydrogel constructs promotes chondrogenic tissue development through induction of transforming growth factor-β1 (TGF-β1) synthesis [36]. Applied deformational loading [37] can also affect the distribution of MSC-elaborated cartilaginous ECM within the tissue constructs through enhanced convective solute transport [38]. Cyclic substrate deformation inhibited proliferation and enhanced osteogenic differentiation of human MSCs [39]. Cyclic tensile loading of MSCs encapsulated in fibrin, applied in the absence of soluble differentiation factors, was found to enhance the expression of both tenogenic and osteogenic markers, while suppressing markers of adipogenesis. No evidence of chondrogenesis was observed, suggesting that cyclic tensile loading can play a role in initiating direct intramembranous ossification [40]. Cyclic strain of cells in other 3D scaffolds has demonstrated osteogenesis as well [41,42,43].

**Figure 4:**
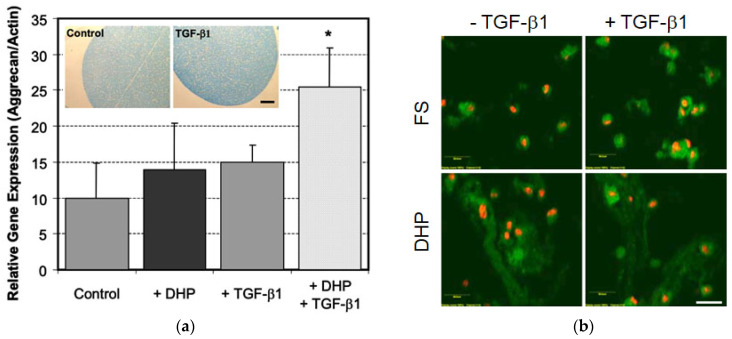
(**a**) Human BMSCs encapsulated in alginate were cultured in chondrogenic media +/− applied dynamic hydrostatic pressure (DHP, 3 MPa, 0.33 Hz, one hour per day) or free-swelling (FS) +/−10 ng/ mL TGF-β1. Inset: Alcian blue staining for glycosaminoglycans, scale bar = 250 µm. Aggrecan mRNA levels normalized to β-actin on day 14 (* *p* < 0.025, n = 3). (**b**) Type II collagen staining (green) with ethidium homodimer stained nuclei (red) on day 14. Scale bar = 50 µm.

Hydrostatic Pressure Loading: Hydrostatic pressures of 300 kPa to as low as 10 kPa, which drive fluid flow within bone, can drive human bone MSC osteogenic lineage commitment [44], where cyclic hydrostatic pressure has been reported to elicit variable, gene-specific responses that are magnitude- and frequency-dependent. Hydrostatic pressure also promotes the proliferation and osteogenic/chondrogenic differentiation of MSCs that is dependent on Ras homolog gene family member A (RhoA) and Ras-related C3 botulinum toxin substrate 1 (Rac1) [45]. Even in the absence of growth factors/cytokines, applied deformational loading and corresponding hydrostatic pressurization leads to loading-induced proteoglycan synthesis [46]. Our own studies have demonstrated that applied cyclic hydrostatic pressure induces aggrecan gene expression and corresponding cartilaginous matrix elaboration that can be enhanced by TGF-β1, as shown in Figure 4a,b.

Fluid Flow: Short periods of mechanical stimulation in the form of oscillatory fluid flow is sufficient to enhance osteogenic gene expression and proliferation of human MSCs [19]. Bone tissue engineering using MSCs is facilitated via perfusion bioreactors that provide fluid shear and nutrient flow [47]. A bioreactor that provides parallel chondrogenic and osteogenic media perfusion to bilayered MSC-scaffold seeded constructs successfully promotes development of osteochondral constructs [48]. Synovial MSCs are mechanosensitive to fluid shear—possibly sensed via primary cilia, with signal transmitted intercellularly through gap junctional communication [49].

Clinical Translation: The various biophysical stimuli reviewed above can be used to prime cell sources for in vitro cartilage, bone and osteochondral tissue engineering applications, as well as for direct stem cell delivery strategies to patients. With respect to in vivo translation of biophysical stimuli for modulating MSC differentiation and function, PEMF for bone repair has proven to be the most significant clinical modality. Two features have made PEMF a particularly attractive translational tool. It can be applied to patients non-invasively, and it can mobilize patients’ resident stem cells for tissue repair rather than require an external cell source. Accordingly, below we devote a section to explore the regulation of mesenchymal cell differentiation by PEMF which have wide clinical applications.

## 3. Experimental Differentiation of Mesenchymal Cells to Chondrocytes and Osteocytes by PEMF In Vivo

Most likely, in vivo populations of what are known as MSCs in various tissue niches are cells of heterogeneous sources and types. For example, in bone, osteoblasts may differentiate from several populations of multipotent cells including endosteal MSCs, periosteal stem cells, angiopericytes, and circulating progenitor cells [50]. They require biochemical or biophysical cues to adopt a lineage commitment and synthesize respective ECM. In parallel with tissue-scale loading, these heterogeneous populations of multipotential stem cells are subject to a wide range of endogenous biophysical cues within their native in vivo microenvironments—notably, mechanical stress. Mechanical regulation can be transduced through the movement of fluid via hydrostatic pressure, fluid shear stress, streaming potentials, and other electrokinetic events that influence lineage commitment [9]. Phenotypic expression of skeletal cells in vivo can also be influenced by externally applied mechanical, electrical, and electromagnetic fields. In particular, it appears that all of these related biophysical phenomena can open voltage-activated transmembrane cation channels leading to calcium flux and signaling. This section will explore the regulation of mesenchymal cell differentiation in a developmental context in endochondral bone formation by external PEMF which have wide clinical applications in the stimulation of skeletal repair.

Because of the intimate linkages of mechanical strain-related fluid flow and electrokinetic phenomena that may serve as secondary cellular messengers, the exposure of experimental systems to isolated electrical or electromagnetic stimuli offer the opportunity to study the effects of these cell signals distinct from mechanics, fluid flow, or other biophysical properties. Exposure of bone marrow-derived MSCs to a rectangular 7.5 Hz PEMF results in the accelerated onset of osteogenesis compared to unexposed controls, as assessed by alkaline phosphatase activity and Runx2/Cbfa1, a key transcription factor for osteogenesis [51]. In another study with human bone marrow-derived MSCs, a 75 Hz square wave PEMF was explored for its effects on osteogenesis measured by alkaline phosphatase and osteocalcin production [52]. Both alkaline phosphatase and osteocalcin synthesis were enhanced by exposure to electromagnetic fields. PEMFs have also been shown to enhance osteogenesis of MSCs when combined with BMP-2 [53]. A series of studies has shown that PEMFs enhance chondrogenesis by increasing the expression of SOX9, aggrecan, and collagen II genes by MSCs of various sources including umbilical cord- and adipose-derived cells [54]. The responses appear to be signal specific [34]. PEMF exposure has also been shown to enhance chemotaxis and MSC migration, an important demonstration in the light of the endochondral bone response in the decalcified bone matrix model discussed below and its enhancement by PEMF. The effects of appropriately configured electromagnetic fields on MSCs for cartilage repair include chemotaxis for MSCs and chondrogenic differentiation of MSCs through a TGF-β mechanism [55]. In bone, PEMF exposure stimulates the expression of osteogenic genes—notably, alkaline phosphatase and osteocalcin [56,57].

The external exposure of MSCs to electric fields stimulates transmembrane calcium channels and affects cell differentiation and ECM expression [58]. Exposure to PEMF has several transmembrane effects. Some studies have shown stimulation of transmembrane calcium signaling [59]. Other studies have shown robust effects upon adenosine receptor signaling and have identified adenosine receptors as the main target of PEMF stimulation [60,61]. Electromagnetic field exposure induces a significant increase in A_2A_ and A_3_A receptor density on the cell membrane of chondrocytes, synoviocytes, and osteoblasts, and influences the microenvironment of MSCs by antagonizing the inhibitory effects of IL-1 and synergizing with TGF-β, inducing chondrogenic differentiation [62].

A systematic review of PEMF exposure of MSCs describes inconsistent effects on *cell proliferation*, not surprising given the wide variety of signals applied, the varied positions in the cell cycle during exposure, and the different management of cell conditions including plating density in vitro [63]. On the other hand, PEMF exposure appeared to predictably increase *cell differentiation* toward both chondrogenic and osteogenic lineages. Bone-specific markers, including BMP-2, TGF-β, and osteocalcin among others, are enhanced by PEMF exposure in the early phases of differentiation and not in the later stages of mineralization; observations that we will see below are replicated in in vivo studies of endochondral ossification.

### 3.1. Electromagnetic Stimulation of In Vivo Mesenchymal Cell Differentiation, Chondrogenesis, and Osteogenesis

Advantages of in vivo studies with a highly inbred strain of animals are the avoidance of artifacts caused by reductionist tissue culture, relative consistency of cell cycle during exposures, maintenance of cells and intercellular relationships within the ECM, and the ability to observe developmental processes, such as endochondral ossification, that are translationally relevant to human pathophysiology.

**Figure 5:**
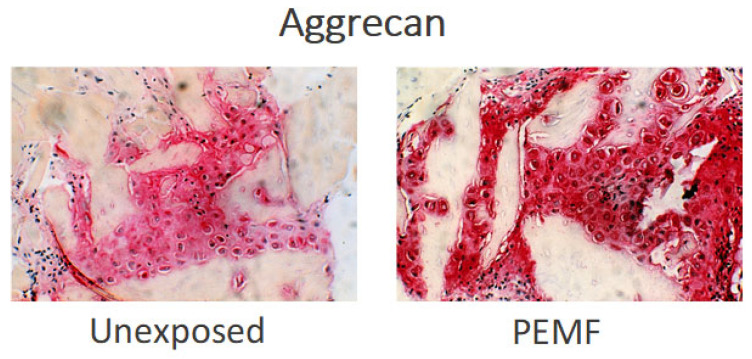
Immunohistochemistry of aggrecan during chondrogenesis (day 8 of ossicle development). Gray areas are DBM particles; red areas represent aggrecan. Ossicles exposed to PEMF stimulation during development form a greater amount of aggrecan than the unexposed controls, as demonstrated by increased areas and intensity of aggrecan staining [65].

**Figure 6:**
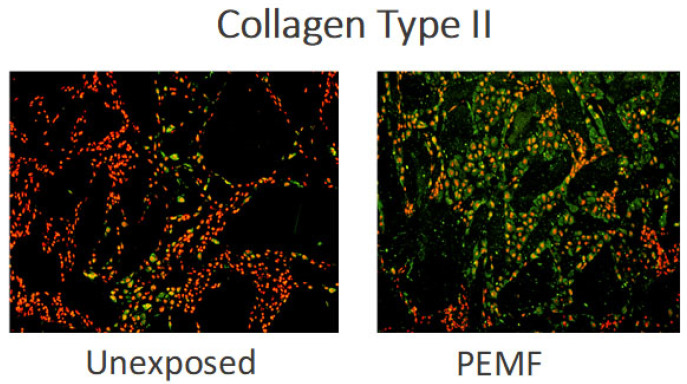
Fluorescein immunohistochemistry of type II collagen during chondrogenesis (day 8 of ossicle development). Black areas are DBM particles. Nuclei are stained red with propidium iodide. Fluorescein stained type II collagen ECM and chondrocytes (green) are more prominent in PEMF-exposed ossicles compared to unexposed ossicles [65].

The developmental nature of endochondral bone formation permits the examination of the effects of PEMF on both the timing and magnitude of cell differentiation and ECM formation of cartilage and bone. To achieve this, the model of demineralized bone matrix (DBM)—induced endochondral ossification described by Marshall Urist and A. Hari Reddi—has been used. In this model, the subcutaneous implantation of DBM powder along the ventral thoracic musculature of CD rats results in a temporally predictable sequence of mesenchymal cytotaxis on days 2–4 after DBM implantation, chondrogenic differentiation by day 6–10, endochondral calcification on days 10–12, and ossification to a mature ossicle with trabecular bone, cortex, and marrow elements by days 14–21 after DBM implantation [64]. The predictable temporal sequences of cell differentiation and ECM synthesis make this model highly suitable to study the effects of stimuli upon the cell biology of endochondral ossification and its amplification. The DBM model has been used to explore the effects of applied electromagnetic energy in the form of PEMF upon DBM-induced endochondral ossification, particularly upon cell differentiation and ECM synthesis. The details of DBM production and implantation, and the PEMF stimulation, have been reported several times [64,65]. Figure 5 and Figure 6 show the relative formation and maturation of cartilage ECM under PEMF exposure compared to endochondral ossification in unexposed animals.

**Figure 7:**
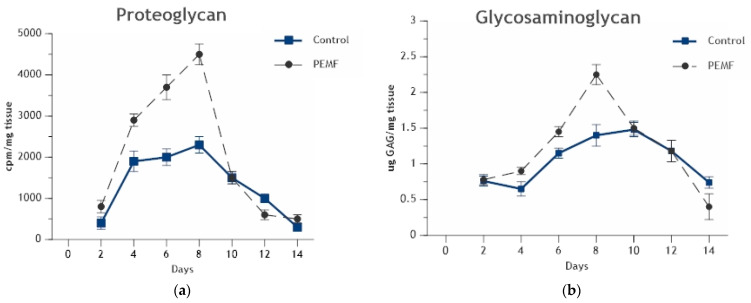
(**a**) Incorporation of radiolabeled sulfate into proteoglycan. Ossicles exposed to PEMF displayed a significant increase in proteoglycan synthesis by day 4 of ossicle development, peaking during maximal chondrogenesis on day 8 (*p* = 0.001) and falling to control levels coincident with the onset of calcification. (**b**) Glycosaminoglycan content. Ossicles exposed to PEMF exposure exhibit increases in glycosaminoglycan content during chondrogenesis (days 4–8) (*p* = 0.05 on day 6 and *p* = 0.01 on day 8). With the onset of calcification on day 10, cartilage matrix is resorbed in both control and PEMF-exposed ossicles [64].

PEMF Stimulation of Endochondral Ossification: Radiolabelled sulfate incorporation and glycosaminoglycan content have demonstrated an accelerated formation and significant increase in chondroid matrix in PEMF-exposed ossicles, compared to control, without disturbing the important cessation of chondrogenesis necessary for calcification (Figure 7a,b) [64,65]. Under PEMF exposure, chondrogenesis ceases on schedule and cartilage matrix is resorbed at a time appropriate to the onset of calcification and bone formation to occur. An acceleration of endochondral calcification and an increased maturation of trabeculae formation are observed in PEMF-treated ossicles [66].

Locus of the PEMF Differentiative Stimulus: Periodic PEMF exposure has been utilized to determine the locus of response, or developmental stages of endochondral bone that are sensitive to electromagnetic stimulation and contain a progenitor cell population competent to respond. Observations of cartilage and bone differentiation suggest that there are differing sensitivities to PEMF stimulation among the developmental stages, with the stage of greatest sensitivity during the mesenchymal stage. During this stage, cell recruitment and differentiation occur. Studies of DNA content and ^3^H thymidine incorporation indicate that proliferation most likely does not occur and, together with the earlier appearance and greater accumulation of cartilage matrix and chondrocytes, the aggregate observations suggest that chondrogenic and osteogenic differentiation are enhanced [66].

**Figure 8:**
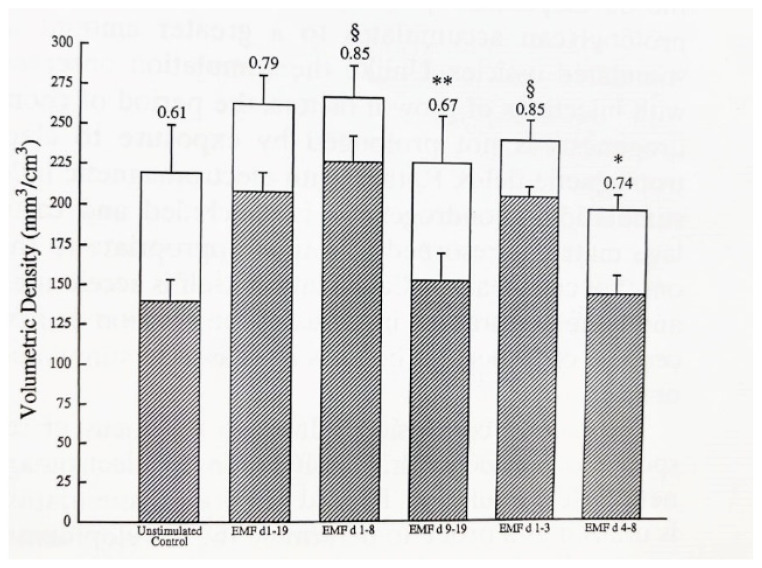
Histological measurement of calcified tissue (open bars) and mature trabeculae (hatched bars). The fraction of total calcified tissue comprised of trabeculae is a measure of ossicle maturation. Ossicles exposed to PEMF during the mesenchymal (days 1–3) or mesenchymal and chondrogenic phases (days 1–8) have an amount of calcified tissue and trabecular maturation equivalent to that of ossicles exposed throughout the full development sequence (day 20). Exposure of ossicles to PEMF at other developmental stages did not induce maturation as compared with stimulation on days 1–19. § = not significant, * *p* < 0.02, and ** *p* < 0.01 [66].

Ossicles exposed during the mesenchymal and chondrogenic phases (days 1–8) or the mesenchymal phase (days 1–3) exhibit trabecular volume and maturation equivalent to that seen in ossicles stimulated throughout their developmental sequence (Figure 8) [66]. Ossicles stimulated during chondrogenesis or bone formation only did not exhibit more bone formation or maturation. These observations indicate that exposure to PEMF during the mesenchymal phase of endochondral bone formation increases the downstream maturation of trabecular bone.

Measurements of chondrogenesis on day 8 of ossicle development have demonstrated that exposure to PEMF during the mesenchymal or chondrogenic phases stimulate indices of chondrogenesis similarly (Table 1) [67]. Thus, it appears that exposure to PEMF during the mesenchymal stage produces an increase in both bone and cartilage formation, whereas exposure during the cartilage phase increases chondrogenesis but not the formation of trabecular bone. These observations collectively suggest that the mesenchymal cell pool is most responsive to PEMF exposure for enhanced bone formation.

These observations suggest several effects of PEMF in this developmental model of endochondral bone formation. Cartilage matrix proteoglycan appears earlier in the developmental process and accumulates to a greater amount in implants exposed to PEMF. Chondrogenesis is concluded and cartilage matrix is resorbed at a time appropriate to the onset of calcification and bone formation. The amount and maturation of bony trabeculae are increased in PEMF-exposed ossicles. Stimulation during the chondrogenic stage only, while sufficient to increase cartilage formation, is insufficient to increase trabecular bone formation. Increasing chondrogenesis without stimulating the early progenitor cell pool does not result in an increased amount of bone. Conversely, PEMF exposure during the mesenchymal stage increases chondrogenic differentiation, cartilage ECM formation, calcification, and trabecular maturation to a degree not observed with stimulation of other stages of endochondral bone formation. This localizes the locus of response to cells in the mesenchymal stage that is competent to respond to electromagnetic field stimulation and to subsequently result in enhanced chondrogenesis and more mature trabecular bone formation.

Clinical Translation: PEMF exposure enhances cartilage and bone formation but, unlike some growth factors, does not disorganize endochondral ossification. These observations have supported the use of PEMF to enhance bone repair in clinical situations, prominently in fracture non-unions. Controlled studies have demonstrated the efficacy of PEMF in enhancing deficient bone repair. A recent review has focused on the role of signal transduction in PEMF efficacy and has described controlled studies of PEMF-stimulated bone repair. Anabolic effects of PEMF on articular cartilage are described as well [68]. The biologic responses of engrafted MSCs to PEMF have not been described but hold substantial theoretical promise.

### 3.2. A Potential Intermediary Mechanism of Tissue Stimulation by Electromagnetic Exposure

This review has emphasized the direct effects of biophysical stimuli on cells. There are a number of potential intermediary mechanisms of action between biophysical agents and cellular recognition and responses. The stimulation of growth factor synthesis by PEMF and other biophysical stimuli has been the subject of several reviews and will not be addressed here so as not to be duplicative [69]. Within the last few years, interactions between vascular cells and MSCs have been recognized for their regulation of bone morphogenesis. In particular, endothelial cells are important paracrine signaling structures that can serve to regulate tissue morphogenesis.

Endothelial cells are part of MSC niches that play key roles through paracrine signaling in regulating a number of processes culminating in bone formation [70]. Bone homeostasis is comprised of coupled osteoblastic new bone formation and osteoclastic bone resorption and resident hematopoietic stem cells as well as endothelial cells play key regulatory roles in the balance of skeletal maintenance. In embryogenesis and endochondral bone repair, vascular invasion of calcifying cartilage brings with it endothelial and hematopoietic stem cells as well as osteoblast and osteoclast precursors which, together, replace the cartilage anlage eventually with trabecular bone and complete the morphogenesis of bone formation and fracture repair. Reciprocal regulation of endothelial cells is provided by the pro-angiogenic cytokine, vascular endothelial growth factor (VEGF) elaborated from chondrocytes. Blood vessels in the skeleton are functionally specialized and bone vasculature is composed of various vessel subtypes that differentially regulate osteogenesis and hematopoiesis [71]. Some endothelial cells (type H) express the pro-osteogenic factors, CD31, PECAM 1, and endomucin, and promote osteoprogenitor cells at sites of endochondral bone formation. Reciprocally, signaling by Notch and hypoxia-inducible factor (HIF) enhance the expansion of type H-containing vessels [72]. This reciprocity of stimulation is reflected in a symbiotic coupling of angiogenesis and osteogenesis. Finally, the important morphogens, bone morphogenic proteins (BMP), stimulate both angiogenesis and osteogenesis. A recent review described the distribution of vascular subtypes, their cytokine expression, and their functions in bone [71]. While a detailed reiteration of this review would be redundant, the review does summarize important bidirectional relationships of cytokines in angiogenesis and skeletogenesis through reciprocal molecular crosstalk between endothelial cells and cells in the skeletal lineage. HIF is a transcriptional regulator that stimulates both neo-angiogenesis and chondrogenesis. VEGF is an important regulator of angiogenesis and cells in the skeletal lineage, including hypertrophic chondrocytes. Osteoblasts and osteoclasts secrete VEGF and influence the proliferation of endothelial cells and the morphogenesis of hypertrophic cartilage, bone formation, and angiogenesis [71]. Other cytokines of importance in the MSC microenvironment are fibroblast growth factor (FGF) and platelet-derived growth factor (PDGF), expressed by both endothelial cells and osteoclast precursors and stimulate migration, proliferation, and differentiation of MSCs in support of both osteogenesis and angiogenesis. These reciprocal molecular interactions are of importance in the physiological processes of both skeletal morphogenesis and bone repair.

PEMF has been used extensively in the clinical enhancement of skeletal repair and its tissue-based mechanisms have recently been reviewed [68]. The cytokine-mediated relationship between angiogenesis and fracture repair has been well described [70]. It is not clear if the stimulation of angiogenesis is a major intermediary mechanism of PEMF action on skeletal repair but PEMF stimulation of angiogenesis is manifested by increased vascular growth rate and capillary density related to endothelial cell proliferation [73]. Because of these observations, PEMF exposure is described here as a model of biophysical stimulation of angiogenesis consistent with its well-described clinical and pre-clinical effects on bone cells and skeletogenesis in the setting of bone repair. Enhancement of vascular morphogenesis by PEMF has been associated with increased expression of VEGF, FGF2, and angiopoietin [73]. Other studies have also reported that PEMF exposure stimulates angiogenesis most likely through an FGF-mediated mechanism [74,75]. PEMF has been shown to have its actions on several cell types including hematopoietic and skeletal through adenosine A2A receptors as described above and reviewed elsewhere [68]. Therefore, it is of interest that the activation of A2A receptors is essential for angiogenesis and secretory products of macrophages during bone healing and that the inactivation of adenosine A2A receptors results in both deficient angiogenesis and fracture nonunion [76].

## 4. Discussion

This review has presented a variety of responses of MSCs to biophysical stimuli, primarily mechanical, electrical, and electromagnetic. MSCs and these biophysical forces enhanced tissue repair—notably, skeletal repair through endochondral bone formation. Understanding the responses of MSCs to their physical environment and to externally applied physical forces, therefore, is important in order to develop optimized therapeutic physical modalities.

The sources of MSCs and influences upon their lineage commitments are also of substantial importance in developing repair strategies. While lineage tracing has provided some clues regarding the developmental origin of MSCs [77,78], the long-established paradigm that they arise from stroma, the supportive connective tissue surrounding organs, has shifted to that of a vascular tissue origin [79,80]. Together with clinical evidence that has suggested that the primary role of injected MSCs in tissue repair is their modulation of the immune system [81], Dr. Arnold Caplan, who initially coined the term MSC, has advocated for renaming them “medicinal signaling cells” that “home in on sites of injury or disease and secrete bioactive factors that are immunomodulatory and trophic (regenerative) meaning that these cells make therapeutic drugs in situ that are medicinal” [82]. In this context, further research on biophysical regulation of MSCs as reviewed herein can help harness their reparative capacity and/or leverage their immunomodulatory role [83,84,85]. In keeping with Caplan’s revised description of MSCs, their potential roles have been expanded to include regulators of tissue homeostasis and modulators in a variety of inflammatory and degenerative systemic diseases via their secretome [86]. MSC paracrine mechanisms displaying proliferative, immunoregulatory, anti-oxidative, or angiogenic activity have been reported in vivo [86]. Conditioned media from MSCs contain several anti-inflammatory and immunomodulatory factors [87], including TGF-β, hepatic growth factor (HGF), indolamine 2,3-dioxygenase-1 (IDO-1), interleukin (IL)-10, IL-1 receptor antagonist (IL-1Ra) and prostaglandin E_2_ (PGE_2_) [84,88,89,90]. MSCs may first prepare the wound milieu for their subsequent tissue repair function by modulating the local inflammatory environment [91].

Applications of MSCs in the context of repair have focused largely on their injection in minimally manipulated autologous cell preparations for which the US Food and Drug Administration (FDA) biologics license approval is not needed [92], including bone marrow aspirate concentrate and adipose or placental tissue fragments [93,94,95]. While the use of such human cells and tissue products requires clinicians to register their use and follow good tissue practice regulations, the regulatory pathway for engineered tissue products is arduous, lengthy and expensive, as approval from the FDA is required when cell isolation, expansion and culture of cells are included in the therapy. Because of FDA requirements, the in vitro priming strategies described in Section 2 to promote chondrogenesis and osteogenesis of MSCs and/or introduction of biophysical and chemical stimuli via bioreactor cultivation [96] would disqualify MSC-derived cartilage and bone constructs from FDA approval. It was only in 2016 that the Autologous Cultured Chondrocytes on Porcine Collagen Membrane (MACI) [97] became the first FDA-approved product that applies the process of tissue engineering to grow cells on scaffolds using healthy cartilage tissue from the patient’s own knee [98]. Despite severe regulatory controls, in vitro techniques for enhancing MSC lineage commitments are being heavily researched. For tissue engineering applications, two-dimensional culture can be used to facilitate priming of MSCs toward a chondrogenic and osteogenic lineage. Primed cells are seeded onto biocompatible scaffolds to form tissue engineered cartilage and bone grafts, respectively [99]. Depending on the clinical need, these engineered tissues may be cultivated to promote their functional development before implantation or implanted for maturation of properties in situ. Acellular scaffolds can also be used to deliver factors, including growth factors or plasmids, which can promote homing and differentiation of resident stem cells [100,101].

Internally or externally manipulated cells, including but not limited to MSCs, are currently under significant FDA scrutiny due in part to the dearth of adequately powered, placebo-controlled studies demonstrating their efficacy [102], unproven advertised claims of stem cell therapies under investigational status, and products that have attempted to expand the definition of minimal manipulation [103]. At the present time, the regulatory landscape encourages the development of therapeutic strategies that can mobilize resident MSCs rather than require their external delivery—notably, strategies aimed at augmentation of marrow stimulation or drilling with stabilizing polymers for cartilage repair [104,105] and application of exogenous electromagnetic stimulation, as described in this review. Many studies have indicated that externally applied electrical and electromagnetic stimuli can affect MSCs from sources other than DBM-induced ossicles. In vitro studies have shown that endochondral bone formation by marrow-derived MSCs can be augmented by exposure to an appropriately configured electrical field [16]. Chondrogenesis was initiated earlier and greater amounts of both cartilage and bone were formed in electrically stimulated cultures. Another study of marrow-derived mesenchymal cells treated with an inductively coupled field demonstrated enhanced ECM synthesis [19]. Fracture callus cells exposed to electrical stimulation just before their proliferative phase increased [^3^H] thymidine incorporation and cell replication [21]. Therefore, in several model systems, proliferation and differentiation of MSCs can be modulated by appropriately applied biochemical or biophysical stimuli. We have described here several studies showing the enhancement by PEMF of the time course and magnitude of trabecular bone formation in an in vivo developmental model of endochondral bone formation. These studies suggest that the pool of responsive cells reside in the early mesenchymal phase of cell recruitment and result in accelerated and amplified cartilage and bone formation. The clinical results of stimulation of resident endogenous cells with external electromagnetic fields have been so successful that these devices are in wide clinical use. According to the Global Market Insights Report, the Bone Growth Stimulators Market exceeded USD 875M in 2021, of which, the growth stimulation devices segment exceeded USD 437M. Prescriptions for the bone growth stimulator market alone were between 125–150 K in 2021 [106]. This is due primarily to the patient’s increasing knowledge of bone growth stimulation devices and expanding technological innovations. Furthermore, the spinal fusion surgery segment is anticipated to see a significant increase, reaching more than USD 461M by 2028 [106].

The study of mechanobiology and electrobiology in orthopedics—in both basic and translational science—can also inform therapies and pharmaceutical interventions in clinical medicine [107]. For instance, the discipline of rehabilitation medicine utilizes a variety of physical stimuli—including electrical, thermal, and mechanical stimulation—effectively in the clinic [108]. Clinical application of PEMF can be optimized through efforts to uncover the mechanisms of the effects of PEMF on chondrogenic and osteogenic lineage commitment of MSCs, which have suggested that PEMFs influence the cellular microenvironment in two ways—stimulating chondrogenic and osteogenic gene expression and suppressing inflammatory and degradative cytokines. Agents that target mechanosensitive channels, such as Transient Receptor Potential Cation Channel Subfamily V Member 4, may provide new drugs to modulate cellular activities in conjunction with physical loading [109].

This review has integrated in vitro and in vivo evidence of effects of biophysical forces upon MSCs that may be useful in advanced therapeutics for tissue repair. The physical microenvironment of MSCs has been described as well as the responsiveness of the cells to mechanical and strain-associated forces. Using MSCs in a therapeutic context to effect repair requires understanding of their complex communication mechanisms. The “language” of the MSCs most likely involves a complex of biochemical and biophysical signals that are integrated with each other and, in a developmental analogy, may require the receptivity of the MSCs to respond to extracellular signaling patterns. Therapeutic biophysical signals will have to be tuned to maximal efficiency to achieve optimal dosing and utilize the incredible promise of appropriately harnessed MSCs in the context of repair. 

## Figures and Tables

**Table 1:** Indices of Chondrogenesis with PEMF Exposure [67].

	Control	PEMF	Percent	*p*
**mRNA aggrecan**	6.1	22.5	269	0.02
**mRNA type II collagen**	11.8	21.9	86	0.05
**^35^SO_4_ incorporation (cpm/mg)**	2166 ± 387	4448 ± 293	105	0.005
**GAG content (µg/mg)**	1.4 ± 0.2	2.5 ± 0.2	79	0.01
**Cartilage area (mm^2^)**	24 ± 2.1	148 ± 11.7	517	0.001
**Chondrocytes (n)**	701 ± 227	3582 ± 675	411	0.005
**Chondrocytes/cartilage**	29.2	24.2		n.s.
